# Isoalantolactone: a review on its pharmacological effects

**DOI:** 10.3389/fphar.2024.1453205

**Published:** 2024-09-23

**Authors:** Guang Yang, Longfei Yang, Fei Xu

**Affiliations:** ^1^ Department of Traditional Chinese Medicine, The Second Hospital of Jilin University, Changchun, China; ^2^ Jilin Provincial Key Laboratory on Molecular and Chemical Genetics, The Second Hospital of Jilin University, Changchun, China; ^3^ Department of Acupuncture and Moxibustion, The Second Hospital of Jilin University, Changchun, China

**Keywords:** isoalantolactone, anti-inflammatory, antitumor, antimicrobial, neuroprotection

## Abstract

Isoalantolactone (ISA) is a sesquiterpene lactone that could be isolated from *Inula helenium* as well as many other herbal plants belonging to Asteraceae. Over the past 2 decades, lots of researches have been made on ISA, which owns multiple pharmacological effects, such as antimicrobial, anticancer, anti-inflammatory, neuroprotective, antidepressant-like activity, as well as others. The anticancer effects of ISA involve proliferation inhibition, ROS overproduction, apoptosis induction and cell cycle arrest. Through inhibiting NF-κB signaling, ISA exerts its anti-inflammatory effects which are involved in the neuroprotection of ISA. This review hackled the reported pharmacological effects of ISA and associated mechanisms, providing an update on understanding its potential in drug development.

## 1 Introduction

**FIGURE 1:**
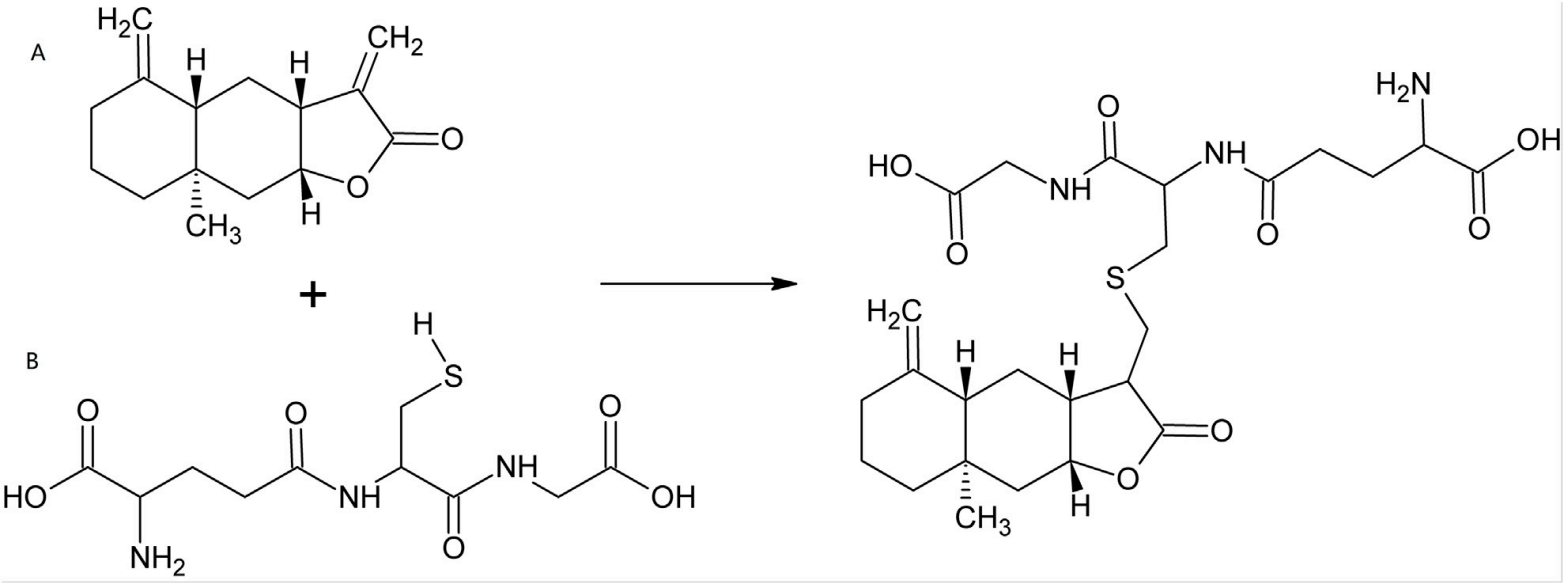
Micheal-type addition of ISA with GSH. **(A)** ISA; **(B)** GSH.

Traditional Chinese Medicine (TCM), including Chinese herbs, has helped people to fight against multiple diseases for thousands of years (Matos et al., 2021). Over the past decades, small molecules deriving from herbal plants, as well as their derivatives, attracted increasing scientific interests in the fields of cancers, inflammations and infections, as well as other diseases (Cantrell et al., 2010; Lawrence et al., 2001; Ma et al., 2024a). Isoalantolactone (ISA, CAS No.: 470-17-7), an eudesmane-type sesquiterpene lactone (Gierlikowska et al., 2020a; Lee et al., 2016), is such a natural compound that exists in the leaves and roots of many plants. Its chemical structure is shown in Figure 1A. The natural sources of ISA are tabulated in Table 1. ISA is also one of the active components from many TCM materials and formulas including TuMuXiang (*Radix inulae*, dried roots of *I. helenium* and *Inula racemosa* Hook f.) (Gao et al., 2018), Mongolian medicine prescriptions Roukou Wuwei pills (Wang et al., 2019), and Liuwei Anxiao San (Wenhua et al., 2004), Tibetan medicine Zuozhu Daxi (Qu et al., 2015) and Ershi-wei Chenxiang pills (Hou et al., 2020). Due to its significance in the content and efficacy (Mohan and Gupta, 2017), ISA, together with alantolactone, was used as the chemical markers of *I. helenium* (Gao et al., 2018). Like the conjugation with GSH (Figure 1), ISA (as well as alantolactone) can also be conjugated with the sulfhydryl group of the free amino acid cysteine in liver (Wang et al., 2018b; Zhou et al., 2018). This was considered as the main metabolic pathway (Zhou et al., 2018). The *in vivo* metabolism of ISA also includes oxidation, hydration and dehydrogenation, which produce 11-carboxyl isoalantolactone, 15-hydroxyl isoalantolactone and 5, 6-dehydroisoalantolactone, respectively (Wang et al., 2018b). In addition, ISA *in vivo* can also undergo demethylation (Yao et al., 2017). 46 ISA metabolites from rat bile, urine and feces are identified by Yao et al. through ultra-high performance liquid chromatography (UPLC)-Triple time-of-flight (TOF)- mass spectrometry (MS)/MS. Among them, 34 are novel sulfur-containing compounds, including two dimers ISA_2_-S (13,13′-thiobis (11α,13-dihydroisoalantolactone)) and ISA_2_-SS (13,13′-dithiobis (11α,13-dihydroisoalantolactone)) (Yao et al., 2017). ISA has shown antimicrobial, antitumor, anti-inflammatory and neuroprotective effects, which were reviewed in the following parts.

**TABLE 1:** The natural sources of ISA.

Plants	Family	Parts	References
*Abutilon indicum*	Malvaceae	Aerial parts	Sharma and Ahmad (1989)
*Ambrosia artemisiifolia*	Asteraceae	Leaves, flowers	Taglialatela-Scafati et al. (2012)
*Artemisia feddei*	Asteraceae	Aerial parts	Tan et al. (1992)
*Artemisia afra* Jacq. Ex Willd	Asteraceae	Leaves	Venables et al. (2016)
*Aucklandia lappa* (*Saussurea lappa*)	Asteraceae	Roots	Kumar and Agnihotri (2020), Kumar et al. (2014a)
*Carpesium cernuum*	Asteraceae	Whole plants	Liu et al. (2010)
*Carpesium longifolium*	Asteraceae	Aerial parts	Yang et al. (2003b)
*Euphorbia stracheyi* Boiss	Euphorbiaceae	Whole plants	Yang et al. (2014)
*Flourensia riparia* Grisebach	Asteraceae	Aerial parts	Uriburu et al. (2004)
*Inula cappa*	Asteraceae	Roots	Kalola et al. (2017)
*Inula helenium*	Asteraceae	Roots	Blagojevic and Radulovic (2012), Yan et al. (2020b)
*Inula japonica*	Asteraceae	Aerial parts	Yang et al. (2003a)
*Inula magnifica*	Asteraceae	Roots	Nikonova (1973)
*Inula macrophylla*	Asteraceae	Whole plants	Ma et al. (2024b)
*Inula racemosa*	Asteraceae	Roots	Kalachaveedu et al. (2018), Mohan and Gupta (2017)
*Inula royleana*	Asteraceae	Roots, aerial parts	Bohlmann et al. (1978), Stojakowska et al. (2006)
*Inula salicina*	Asteraceae	Aerial parts	Bohlmann et al. (1978)
*Stevia lucida* Lagasca	Eupatorieae	Aerial parts	Chacon-Morales et al. (2021)
*Telekia speciosa*	Asteraceae	Roots, stems, flowers, leaves	Wajs-Bonikowska et al. (2012)

## 2 Antibacterial activities

Bacterial pathogens, such as *Staphylococcus aureus* and *Klebsiella pneumonia,* are still a major threat to human health in spite of the many antimicrobial agents (Jin et al., 2023; Zhang et al., 2023). ISA showed weak activities against *B. subtilis*, *E. coli*, *Pseudomonas fluorecense*, *Sarcina lentus* and *S. aureus,* with MIC being 100–425 μg/mL (Table 2) (Liu et al., 2001). This was also confirmed by another report in which ISA showed no antibacterial activities below 128 μg/mL against *E. coli* and *Klesiella pneumoniae* (Lu N. et al., 2020). Interestingly, ISA showed no anti-staphylococcal activities in another study as the MIC against five different *S. aureus* strains were above 1,024 μg/mL according to the CLSI recommended microdilution method (Qiu et al., 2011). Although there may be discrepancy over the antibacterial activity of ISA against *S. aureus*, it should be certain that 1–8 μg/mL of ISA should be ineffective to *S. aureus*, and it was this concentration that can suppress the production of α-toxin, which is a critical virulence factor of *S. aureus* (Qiu et al., 2011). In this scenario, ISA could be considered as an anti-virulence lead for treating *S. aureus* infections. Further investigation revealed that ISA can protect mice against *S. aureus* pneumonia (Qiu et al., 2011). It should be noticed that the analog of ISA, alantolactone, can facilitate the uptake of *S. aureus* by macrophages (Gierlikowska et al., 2020b), therefore ISA may probably have the similar activity, which need to be explored in the future. ISA can also enhance the efficacy of penicillin G in multiple β-lactamase-positive *S*. *aureus* strains, even the methicillin-resistant *S. aureus* (MRSA) strains, both *in vitro* and *in vivo*, and the mechanism involves the inactivation of β-lactamase by ISA during protein translation (Zhou et al., 2020).

**TABLE 2:** Antimicrobial activities of ISA.

Species	Activities	Assays and culture conditions	References
*Bacillus cereus*	ZOI: 17 mm	Agar well diffusion, 18–24 h, 37°C	(Lokhande et al., 2007)
*Bacillus subtilis*	MIC: 125 μg/mL	Agar dilution assay, 35°C, 48 h	(Liu et al., 2001)
*Escherichia coli*	ZOI: 14 mm	Agar well diffusion, 18–24 h, 37°C	(Lokhande et al., 2007)
*Escherichia coli*	MIC: 425 μg/mL	Agar dilution assay, 35°C, 48 h	(Liu et al., 2001)
*Klesiella pneumoniae*	ZOI: 15 mm	Agar well diffusion, 18–24 h, 37°C	(Lokhande et al., 2007)
*Mycobacterium tuberculosis*	MIC: 32 μg/mL	Radiorespirometric assay, 37°C, 10 days	Cantrell et al. (1999)
*Pseudomonas aeruginosa*	ZOI: 18 mm	Agar well diffusion, 18–24 h, 37°C	(Lokhande et al., 2007)
*Pseudomonas fluorecense*	MIC: 150 μg/mL	Agar dilution assay, 35°C, 48 h	(Liu et al., 2001)
*Salmonella typhi*	ZOI: 14 mm	Agar well diffusion, 18–24 h, 37°C	(Lokhande et al., 2007)
*Sarcina lentus*	MIC: 150 μg/mL	Agar dilution assay, 35°C, 48 h	(Liu et al., 2001)
*Serratia marcescens*	ZOI: 14 mm	Agar well diffusion, 18–24 h, 37°C	(Lokhande et al., 2007)
*Shigella dysenteriae*	ZOI: 12 mm	Agar well diffusion, 18–24 h, 37°C	(Lokhande et al., 2007)
*Staphylococcus aureus*	ZOI: 13 mm	Agar well diffusion, 18–24 h, 37°C	(Lokhande et al., 2007)
*Staphylococcus aureus*	MIC: 100 μg/mL	Agar dilution assay, 35°C, 48 h	(Liu et al., 2001)
*Staphylococcus aureus* ATCC 25923	MIC: 21.33 (±9.24) μg/mL	Broth microdilution method, 37°C, 16–18 h	Zhou et al. (2020)
*Staphylococcus aureus* (ATCC 29213, ATCC 10832, BAA-1717, 8,325–4, DU 1090)	MIC: >1,024 μg/mL	Broth microdilution method, 37°C, 24 h	Qiu et al. (2011)
*Fungi*			
*Alternaria brassicae*	ED_50_: 190 μg/mL	Spore germination inhibition, 24°C, 20 h	Kataria and Chahal (2013)
*Alternaria triticina*	ED_50_: 220 ppm	Spore germination inhibition, 24°C, 20 h	Kaur and Chahal (2020)
*Aspergillus flavus*	MIC: 50 μg/mL	Agar dilution assay, 25°C, 72 h	Tan et al. (1998)
*Aspergillus niger*	MIC: 50 μg/mL	Agar dilution assay, 25°C, 72 h	Tan et al. (1998)
*Aspergillus niger* V*. Tiegh*	MIC: 1,500 μg/mL	Mycelial growth inhibition, 25°C, 6 days	(Liu et al., 2001)
*Candida albicans*	MIC: 25 μg/mL	Agar dilution assay, 25°C, 72 h	Tan et al. (1998)
*Candida albicans* DSY654	MIC: 16 μg/mL	Broth microdilution method, 35°C, 48 h	Li et al. (2017)
*Candida albicans* Berkh	MIC: 1,000 μg/mL	Mycelial growth inhibition, 25°C, 6 days	(Liu et al., 2001)
*Candida tropicalis*	MIC: 25 μg/mL	Agar dilution assay, 25°C, 72 h	Tan et al. (1998)
*Dreschlera oryzae*	ED_50_: 200 ppm	Spore germination inhibition, 24°C, 20 h	Kaur and Chahal (2020)
*Fusarium graminearum Schw*	MIC: 1,500 μg/mL	Mycelial growth inhibition, 25°C, 6 days	(Liu et al., 2001)
*Fusarium moniliforme*	ED_50_: 235 ppm	Spore germination inhibition, 24°C, 20 h	Kaur and Chahal (2020)
*Gaeumanomyces graminis (Sacc.) Arx and Oliver* var. *tritici J. Walker*	MIC: 100 μg/mL	Mycelial growth inhibition, 25°C, 6 days	(Liu et al., 2001)
*Gerlachia nivalis* Gams et Mull	MIC: 1,000 μg/mL	Mycelial growth inhibition, 25°C, 6 days	(Liu et al., 2001)
*Geotrichum candidum*	MIC: 25 μg/mL	Agar dilution assay, 25°C, 72 h	Tan et al. (1998)
*Helminthosporium sativus Pam. King et Bakke*	MIC: 1,000 μg/mL	Mycelial growth inhibition, 25°C, 6 days	(Liu et al., 2001)
*Penicillium notatum* Westl	MIC: 1,000 μg/mL	Mycelial growth inhibition, 25°C, 6 days	(Liu et al 2001)
*Penicillium italicum*	ED_50_: 210 μg/mL	Spore germination inhibition, 24°C, 20 h	Kataria and Chahal (2013)
*Penicillium italicum* Wehmer	MIC: 1,500 μg/mL	Mycelial growth inhibition, 25°C, 6 days	(Liu et al., 2001)
*Phytophthora capsici* Leon	MIC: 300 μg/mL	Mycelial growth inhibition, 25°C, 6 days	(Liu et al., 2001)
*Rhizoctonia cerealis* Vander Hoeven	MIC: 100 μg/mL	Mycelial growth inhibition, 25°C, 6 days	(Liu et al., 2001)
*Rhizoctonia solani*	ED_50_: 300 μg/mL	Mycelial growth inhibition, 24°C, 6 days	Kataria and Chahal (2013)
*Trichophyton mentagrophytes* Blanch	MIC: 1,500 μg/mL	Mycelial growth inhibition, 25°C, 6 days	(Liu et al., 2001)
*Trichophyton rubrum*	MIC: 1,500 μg/mL	Mycelial growth inhibition, 25°C, 6 days	(Liu et al., 2001)
*Viruses*			
Vesicular stomatitis virus	IC_50_: ∼5 μM	Viral protein expression, 37°C, 16 h	Kong et al. (2024)
influenza A virus (H1N1)	IC_50_: <10 μM	Viral mRNA replication, 37°C, 8 h	Kong et al. (2024)
Encephalomyocarditis virus	IC_50_: <10 μM	Viral mRNA replication, 37°C, 8 h	Kong et al. (2024)

Abbreviations: MIC, minimal inhibitory concentration; ZOI, zone of inhibition; ED_50_, the median effective dose.

Although ISA showed no or weak antibacterial activities against Gram-negative bacteria *E. coli* and *K. pneumonia*, ISA was proved to be an inhibitor of MCR-1, which remodels the structure of lipids in bacterial LPS diminishing the efficacy of cationic peptide antibiotics such as colistin (Feng et al., 2022; Lu N. et al., 2020; Liu et al., 2001). Therefore, this compound can synergize with polymyxin B or colistin in suppressing the growth of MCR-1 positive *E. coli* and *Klebsiella* strains *in vitro,* and in inhibiting the pathogenicity of these two bacterial pathogens in mice (Lu N. et al., 2020). The mechanisms involve the inhibition of the enzyme activity rather than the production of MCR-1 by ISA, restoring the efficacy of carbapenems (Lu N. et al., 2020).

**FIGURE 2:**
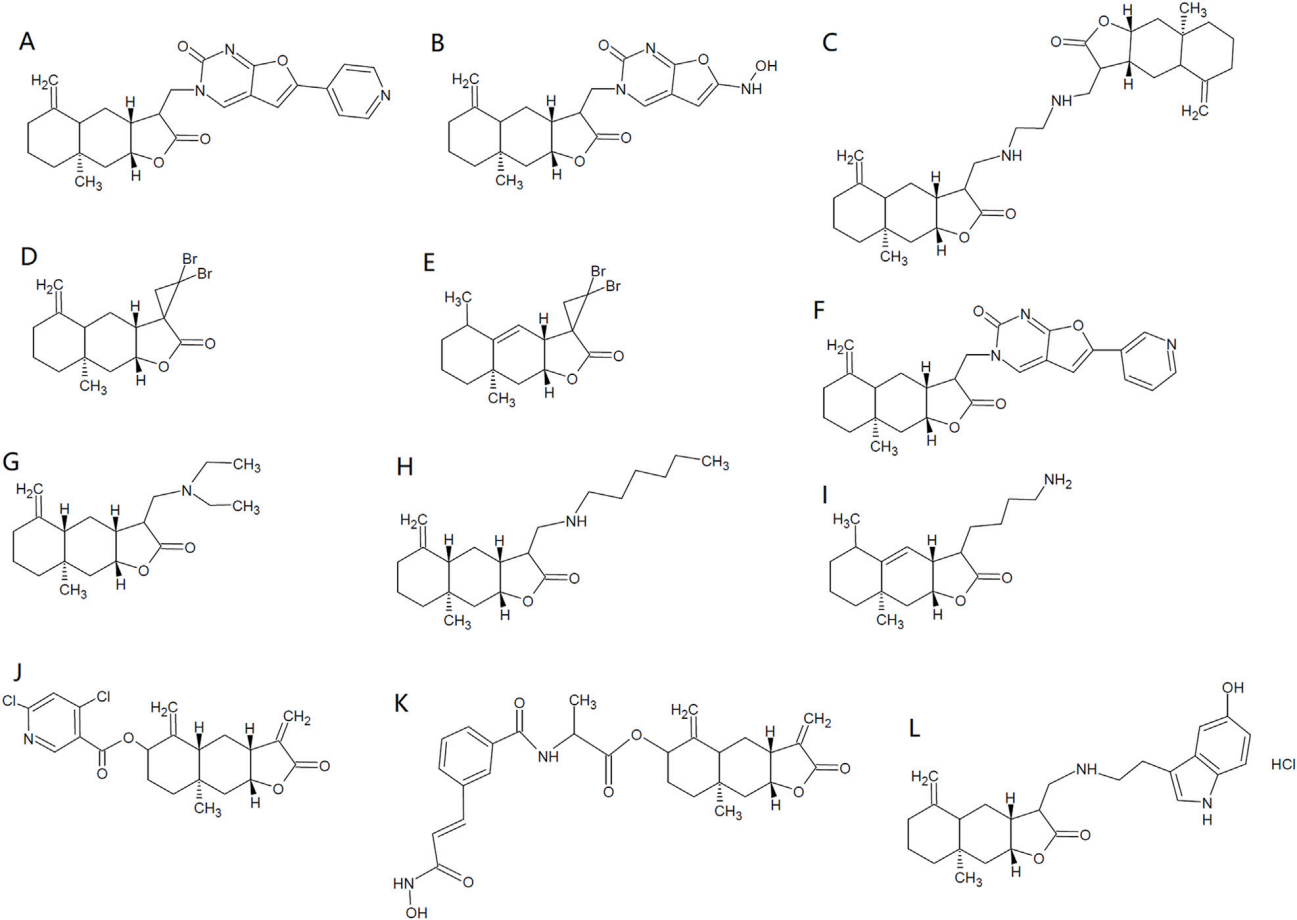
Chemical structures of some ISA derivatives. **(A–L)**, ISA derivatives 1∼12.

Tuberculosis resulting from *M. tuberculosis*, is among the major list of global public health threats (Ramakrishnan, 2020). ISA has shown antimycobacterial activity against *Mycobacterium tuberculosis*, with a minimum inhibitory concentration (MIC) of 32 μg/mL (Cantrell et al., 1999). Coinciding with this, ISA-containing (chloroform, methanol and Petroleum ether) extracts from *I*. *helenium* can inhibit the growth of *M. tuberculosis* H37Rv, with a MIC below 100 μg/mL (Tosun et al., 2008). Structural modifications have been made by Patrushev et al. on ISA to explore the antibacterial activities of ISA derivatives (Patrushev et al., 2021; Patrushev et al., 2023). Among these synthesized analogs, Derivative 1 (Figure 2A) has best activity against *E. faecalis* (MIC: 463.3 μg/mL). Derivative 2 (Figure 2B) has best activity against *A. viscosus* (MIC: 96.5 μg/mL) and *Pseudomonas aeruginosa* (MIC: 399.3 μg/mL) and this compound can also inhibit the biofilm formation of *P. aeruginosa* below its MIC (Patrushev et al., 2021). Derivative 3 (Figure 2C) has best antibacterial activity against *S. aureus* (MIC: 14.6 μg/mL), *B. cereus* (MIC: 11.7 μg/mL) and *E. coli* (MIC: 37.5 μg/mL) (Patrushev et al., 2023).

## 3 Antifungal activities

Fungal pathogens can cause loss in crops and human infections (Niu et al., 2020; Yang et al., 2024). The insufficiency of antifungal agents and the emergence of drug resistance necessitate the development of new antifungal drugs (Chen et al., 2023; Liu et al., 2017; Zhang et al., 2024). The earliest report about the antifungal activity of ISA was published in 1998, where ISA has shown antifungal effects on *Aspergillus flavus*, *Aspergillus niger*, *Candida albicans*, *Candida tropicalis* and *Geotrichum candidum*, with MICs of 25–50 μg/mL (Tan et al., 1998). Later, ISA was confirmed to be effective to inhibit the mycelial growth of *A. niger, C. albicans, Fusarium graminearum, Gacumanomyces graminis*, *Gerlachia nivalis Gams et Mull*, *Helminthosporium sativus*, *Penicillium italicum*, *P. notatum*, *Phytophthora capsica*, *Rhizoctonia cerealis*, *Trichophyton mentagrophytes* and *T. rubrum*, at concentrations ranging from 50–1,500 μg/mL (Liu et al., 2001). Notably, ISA at the concentration of 150 μg/mL (for 48 h), which would kill phytopathogenic fungi, didn`t demonstrate detrimental influence on seed germination and seedling growth of wheat (Liu et al., 2001; Liu et al., 2006). The germination of *Alternaria brassicae* and *Penicilium italicum*, culprits for alternaria blight of rapeseed and blue mould of kinnow, respectively, could be suppressed by ISA, with ED_50_ around 200 μg/mL (Kataria and Chahal, 2013). *Rhizoctonia solani* that causes black scurf and stem canker in potatoes, can also be inhibited by ISA (ED_50_: 300 μg/mL), assayed via poisoned food method (Kataria and Chahal, 2013). ISA also can block the spore germination of phytopathogens *Alternaria triticina*, *Dreschlera oryzae* and *Fusarium moniliforme* (with ED_50_ around 200 ppm), which can reduce the yield and quality of corps (Kaur and Chahal, 2020).

The anti-*Candida* activity of ISA was also confirmed later in a research showing that although the MIC against several *C. albicans* strains were higher than 128 μg/mL, the deficiency of drug efflux pumps significantly increased the anticandidal activity of ISA (Li et al., 2017). And in this deficient strain, ISA can inhibit the hyphal growth, which is critical for *C. albicans* infection (Li et al., 2017; Yang et al., 2022; Zhang et al., 2024). Its antifungal mechanism may involve the elevated accumulation of zymosterol and lanosterol and the reduced ergosterol level, which resulted from inhibition of Erg11 and Erg6 activity (Li et al., 2017). In addition, the essential oils from roots of *I*. *helenium*, which contains both anti-*Candida* alantolactone and ISA, inhibits the growth, hyphae and biofilms of multiple *C. albicans* strains (Stojanović-Radić et al., 2020; Yang et al., 2022). And alantolactone also shows antifungal activities against *C. albicans*, *Candida krusei*, *C. tropicalis* and *Candida glabrata*, with MIC from 18 to 72 μg/mL (Yang et al., 2022). The synthetic ISA analog with the best antifungal activity against *A. triticina* (ED_50_: 155 ppm), *D. oryzae* (ED_50_: 140 ppm) and *F. moniliforme* (ED_50_: 168 ppm) is Derivative 4 (Figure 2D) (Kaur and Chahal, 2020). The alantolactone analog with best antifungal activity against *A. triticina* (ED_50_: 160 ppm), *D. oryzae* (ED_50_: 120 ppm) and *F. moniliforme* (ED_50_: 180 ppm) is Derivative 5 (Figure 2E) (Kaur and Chahal, 2020). They are both better than the parent compounds. Although pyrazolines of ISA also been synthesized, but the antifungal activities against *A. brassicae*, *R. solani* and *P. italicum* were weaker than ISA (Kataria and Chahal, 2013). The antibacterial and antifungal activities of ISA were summarized in Table 2.

## 4 Antiviral activity

Viral infections claim millions of deaths and the need for antiviral agents is increasing as new viruses and antiviral resistance emerge. Vesicular stomatitis virus (VSV), influenza A virus (H1N1) and encephalomyocarditis virus (EMCV) are such viruses that impact public health. One of the ISA sources, *S. lappa* roots, has been employed for treating viral infections in traditional medicine (Kumar et al., 2014a), suggesting the antiviral activity of *S. lappa* components. The protection against herpes simplex virus 1 (HSV-1) provided by the analog of ISA further indicates the presence of ISA antiviral activity (Liu X. et al., 2021). Recently, ISA (added either before or after adsorption), at concentrations nontoxic to human A549 lung cancer cells which are usually used also as models of viral infections, has been reported to significantly inhibit the intracellular replication of VSV and decrease the level of VSV-G protein, despite that ISA has no influence on the adsorption of virus to cell surfaces. In addition, ISA can also inhibit the replication of other viruses, such as influenza A virus (H1N1) and EMCV (Kong et al., 2024). The activation of type I interferon pathway by ISA may further contribute to the suppressing effects of ISA on viral infections (Kong et al., 2024). Moreover, chemical modification of ISA with a furo [2,3-*d*]-pyrimidin-2-оne moiety produce Derivative 6 (Figure 2F) (3-{[(3R,3αR,4αS,8αR, 9αR)-8α-methyl-5-methylene-2-oxododecahydronaphtho [2,3-b] furan-3-yl] methyl}-6-(pyridin-3-yl) furo [2,3-d] Pyrimidin-2(3H)-One), which could enhance the antiviral activity against human orthopneumovirus H-2 (IC_50_: 3.7 μM) while lower the cytotoxicity of ISA (Selective Index >33), highlighting the potential of chemical structural optimization of ISA in developing antiviral drugs (Patrushev et al., 2021).

## 5 Insecticidal activity

The insecticidal activity of ISA was first demonstrated in 2006 by Liu et al., who found that ISA was repellent and toxic against rice weevil (*Sitophilus oryzae*) that damages stored grains. However, the insecticidal was week, with the half maximal lethal concentration (LC_50_) of 707.21 and 610.51 μg/g on 10 and 20 days post-treatment, respectively (Liu et al., 2006). Although pre-soaking with ISA may influence the seed germination and seedling growth of wheat at high concentration (500 μg/mL), this kind of phytotoxicity can be lowered by reducing the duration of treatment (Liu et al., 2006). ISA can also cause damages to the third and fourth instar of *Aedes albopictus* (the vector for the transmission of dengue and West Nile viruses), with a LC_50_ of 11.9 μg/mL (Konishi et al., 2008). ISA also showed insecticidal activity against *Paratanytarsus grimmii*, with a LC_50_ of 4.1 μg/mL, an allergy-causing cosmopolitan midge that can infest municipal water system (Alexander et al., 1997; Konishi et al., 2008). ISA showed insecticidal activity against *Aedes aegypti*, which transmits dengue virus and yellow fever virus, with the LC_50_ of 10 μg/mL for first instar larvae and 2.28 μg/mosquito for adult insects (Cantrell et al., 2010). In addition, ISA can suppress the growth of the third -instar larvae, reduce the *Spodoptera litura* pupation as well as its adult emergence and adult lifespan, possibly attenuating the loss in cotton, cabbages and cauliflowers caused by this insect. ISA actions through reducing food ingestion and digestion efficiency (Kaur et al., 2017). Higher concentrations of ISA could also lead to malformed pupae and adults, which die too early before maturation (or become intermediates between lavae and pupae) and grow wings with deformities (underdeveloped and crumped), respectively. The malformed adults have also lower survival in face of their natural enemies (Kaur et al., 2017). The insecticidal effects of ISA were summarized in Table 3. Synthetic derivatives of ISA have not shown better activity against *A. aegypti* larvae than ISA and the one with the best larvacidal activity is Derivative 7 (Figure 2G) (LC_50_ = 14.4 μg/mL) (Cantrell et al., 2010). However, Derivative 8 (Figure 2H) has best adulticidal activity (LC_50_ = 1.76 μg/mosquito), better than ISA. In contrast, there are several synthetic analogs (such as Derivative 9, Figure 2I) showing better activity than alantolactone (LC_50_ = 36.2 μg/mL) (Cantrell et al., 2010).

**TABLE 3:** The insecticidal activities of ISA.

Insects	Effects/LC_50_	Insect development stages	Treatment time	References
*Sitophilus oryzae*	707.21 μg/g	Adults	10 days	Liu et al. (2006)
*Sitophilus oryzae*	610.51 μg/g	Adults	20 days	Liu et al. (2006)
*Aedes albopictus*	11.9 μg/mL	3rd and 4th instars	48 h	Konishi et al. (2008)
*Paratanytarsus grimmii*	4.1 μg/mL	3rd and 4th instars	48 h	Konishi et al. (2008)
*Aedes aegypti*	10 μg/mL	1st instar larvae	24 h	Cantrell et al. (2010)
*Aedes aegypti*	2.28 μg/mosquito	adult insects	24 h	Cantrell et al. (2010)
*Spodoptera litura*	Decreased pupation and longevity	3rd instar larvae	∼40 days	Kaur et al. (2017)

## 6 Anti-inflammatory activity

Among its multiple activities, the anti-inflammatory activity is so significant that it has been included in most introductions of publications about this compound. During inflammation, MAPK pathway modulating the proliferation and differentiation of inflammatory cells, and NF-κB signaling pathway regulating the expression of genes of inflammatory mediators, are critical. Although researches on ISA in the field of inflammation did not show the effects of ISA on MAPK, the inhibition of p38 MAPK (Wang et al., 2016), as well as NF-κB, in breast cancers, may probable be extrapolated to inflammatory cells, especially considering that breast cancer is closely associated with inflammation. At the molecular level, ISA is able to directly inhibit the soluble epoxide hydrolase (with an IC_50_ of 63.2 μM), as shown in the soluble epoxide hydrolase-probe hydrolysis assay (He et al., 2020). As the C-terminal epoxide hydrolase of this bifunctional enzyme (located in cytosol and peroxisomes) can catalyze the conversion of epoxy eicosatrienoic acids to toxic and pro-inflammatory vicinal diols, while elevating epoxy eicosatrienoic acids can impose inhibition on NF-κB, the inhibition of soluble epoxide hydrolase by ISA may result in inhibition of inflammation through both sides of the reactions: lowering the pro-inflammatory product and increasing the anti-inflammatory substrate (He et al., 2020; Jonnalagadda et al., 2021).

ISA-containing extract from *I. helenium* not only inhibits the production of NO and PGE_2_ in LPS-activated RAW264.7 murine macrophages, but also attenuates the carrageenan-induced edema in mouse paws, which is a model of acute inflammation (Chun et al., 2019). Another preparation of ISA-containing extract from *I. helenium* can attenuate the atopic dermatitis-like symptoms caused by dinitrochlorobenzene in mice, besides its decrease in the expression of IL-1, IL-4 and TNF-α in TNF-α-stimulated HaCaT cells (Wang Q. et al., 2018).

In LPS-stimulated BV-2 microglial cells, ISA can activate the GSK-3β and its downstream Nrf2 and HO-1, which suppressed the activation of NF-κB and the subsequent production of inflammatory mediators, causing the inhibition of inflammation (Wang et al., 2018a). In another group’s report, ISA inhibits the secretion of pro-inflammatory cytokines from microglial cells exposed to LPS through activating Akt/Nrf2/HO-1 pathway and suppressing NF-κB translocation (He et al., 2022).

The anti-inflammatory effects of ISA were shown not only in cellular assays but also in animal experiments. In LPS-induced acute lung injury of mice, ISA could inhibit PI3K/AKT signaling and upregulate Nrf2/HO-1 pathway, leading to the inhibition of NF-κB signaling. Consequently, the elevation of pro-inflammatory cytokines IL-1β and TNF-α in bronchoalveolar lavage fluid (BALF), as well as neutrophil recruitment, pulmonary edema and alveolar wall thickening caused by LPS was blocked by ISA (Yuan et al., 2018). Later data from another group confirmed its protective effects in acute lung injury and further revealed that ERK phosphorylation (one of the three branches of MAPK pathways) was also suppressed during this process and that of the three proteins regulating NF-κB, the suppression of TNF receptor-associated factor 6 (TRAF6) expression and its polyubiquitination of TRAF6 at lysine 63 (K63-linked polyubiquitination, which may cause IκB kinase (IKK) phosphorylation and activation (Min et al., 2018)), rather than lysine 48, was responsible for ISA-mediated NF-κB inhibition (Ding et al., 2019).

In mouse asthmatic inflammation induced by ovalbumin, ISA treatment can reduce the infiltrated eosinophils (stimulated by CCL17 and CCL22) rather than macrophages, serum ovalbumin-specific IgE, the protein concentration as well as type 2 cytokines (IL-4, IL-5, IL-13, CCL17 and CCL22) in BALF (Song et al., 2021). Further studies revealed that the inhibition of STAT6/PPAR-γ/KLF-4 in BMDM by ISA blocks the alternatively activation of macrophages (M2 type polarization, a main characteristic of asthma inflammation) that is induced by IL-4 (Song et al., 2021). In mice model of sepsis, which is associated with the failures of multiple organs resulting from unconstrained inflammatory responses, ISA can suppress the NF-κB pathway in liver and reduce the decrease in survival rate caused by LPS (He et al., 2017). In a rat model of arthritis induced by collagen or adjuvant, ISA-containing extract mitigates disease severity (Gao et al., 2017). Even in three inflammation models of zebrafish (induced by CuSO4, tail-cutting, and LPS), ISA showed obvious anti-inflammatory effects via suppressing the recruitment of immune cells (Zhou et al., 2023).

Considering that the analog and isomer of ISA, alantolactone has been found to be a NLRP3 inhibitor (Li et al., 2023), ISA is probably an NLRP3 inhibitor that can mitigate multiple kinds of inflammatory responses. Very recently, ISA was found to be a strong inhibitor of NLRP3, with IC_50_ of 7.86 μM in assays to assess the inhibition on IL-1β release induced by nigericin in THP-1 cells (Zhao et al., 2024). This group also synthesized and identified a far more potent ISA derivative, Derivative 10 (Figure 2J) ((3αR, 4αR, 6R, 8αR, 9αR)-8α-methyl-3,5-dimethylene-2-oxododecahydronaphtho [2,3-b]furan-6-yl 4,6-dichloronicotinate), which inhibits NLRP3 with an IC_50_ of 0.29 μM (Zhao et al., 2024). The detailed anti-inflammatory mechanisms of ISA were tabulated in Table 4.

**TABLE 4:** The anti-inflammatory mechanisms of ISA.

Experimental model	Doses	Inflammatory parameters assessed	Biological effects Mechanisms/actions	References
*In vitro* models				
LPS-activated RAW264.7 cells	0.6, 1.2, 2.4 μg/mL; 2, 4, 8 μM	NO, PGE2, IL-1, MCP-1, IL-6, TNF-α	iNOS↓, COX-2↓, NF-κB activation↓	Chun et al. (2019), Gao et al. (2017), He et al. (2017)
LPS-activated mouse peritoneal macrophages	2, 4, 8 μM	NO, PGE2, IL-6, TNF-α	iNOS↓, COX-2↓, IκKα/β phosphorylation and degradation ↓, IκBα↑, NF-κB activation↓	He et al. (2017)
LPS-stimulated BV2 cells	2.5, 5, 10 μM	IL-1β, TNF-α, NO, PGE2	Activation of NF-κB↓, Nrf2 ↑, HO-1↑, GSK-3β phosphorylation↑	Ma et al. (2024b), Wang et al. (2018a)
LPS-stimulated BV2 cells	1, 2 μM	IL-6, TNF-α, iNOS, COX2	Activation of AKT/Nrf2/HO-1↑, Activation of NF-κB↓	He et al. (2022)
LPS-stimulated BMDMs	2.5, 5, 10, 20 μM	IL-1β, IL-6, TNF-α, NO	TRAF6 polyubiquitination↓, Activation of NF-κB↓, ERK↓, AKT↓, iNOS↓	Ding et al. (2019)
Ethanol-treated GES-1 cells	1.25, 2.5, 5 μM	IL-1β, IL-10, TGF-β1, IFN-γ, TNF-α, VEGFA	Unexplored	Zhou et al. (2023)
Nigericin-induced THP-1 cells	2, 5 μM (IC_50_ = 7.86 μM)	IL-1β release	NLRP3 inflammasome inhibition	Zhao et al. (2024)
High glucose-treated rat mesangial cells	0.2, 1, 10 μM	MCP-1, TGF-β1	Degradation of IκBα↓, NF-κB DNA binding↓	Jia et al. (2013)
TNF-α-stimulated HaCaT cells	0.6, 1.2, 2.4 μg/mL	IL-1, IL-4, TNF-α	IκKβ phosphorylation and degradation ↓, p65 phosphorylation↓	Wang et al. (2018c)
TNF-α-stimulated synovial fibroblasts	0.6, 1.2, 2.4 μg/mL	IL-1, IL-6, iNOS	NF-κB activation↓	Gao et al. (2017)
***In vivo*** **models**				
DSS-induced experimental colitis (mice)	30 mg/kg/day	TNF-α, IL-1β	NLRP3 inhibition↑, caspase 1↓, Disease activity index↓, colon length↑	Zhao et al. (2024)
LPS-induced acute lung injury (mice)	2.5, 5, 10 mg/kg	TNF-α, IL-1β	NF-κB↓, PI3K phosphorylation↓, AKT phosphorylation↓, Nrf2 ↑, HO-1↑	Yuan et al. (2018)
LPS-induced acute lung injury (mice)	20 mg/kg	IL-1β, IL-6, TNF-α	TRAF6 polyubiquitination↓, Activation of NF-κB↓, ERK↓, AKT↓, iNOS↓	Ding et al. (2019)
LPS-induced sepsis (mice)	5, 10 mg/kg	IL-6, TNF-α	Survival rate↑	He et al. (2017)
Ovalbumin-induced asthmatic inflammation (mice)	20 mg/kg/day	IL-4, IL-5, IL-13, CCL-17, CCL-22	IL-4-induced STAT6 phosphorylation↓, PPAR-γ↓, KLF4↓	Song et al. (2021)
CuSO_4_-induced inflammation model (zebrafish)	1.25, 2.5, 5 μM	*tnf-α, ifn-γ, tgf-β1* expression	Immune cells recruitment↓, ROS production↓, PI3K-Akt signaling↓	Zhou et al. (2023)
MPTP-induced PD in mice	10 mg/kg/day	IL-1β, IL-6, TNF-α	Microglial activation↓, dopaminergic neuron degeneration↓, striatal dopamine↑	He et al. (2022)
Chronic social defeat stress-induced depressive behaviors (mice)	10 mg/kg/day	IL-6, TNF-α	BDNF↑, GluA1↑, PSD95↑, depression-like behaviors improved	Wang et al. (2023)

↓, decrease; ↑, increase.

## 7 Antitumor activity

ISA is reported to have significant anticancer effects in various kinds of cancers, including breast cancer (MDA-MB-231, BT-549 and MCF-7) (An et al., 2024; Li et al., 2016; Wu et al., 2016), ovarian cancer (SKOV3, SHIN-3, HOC-21, HAC-2; OVCAR-3 cells) (Liu et al., 2020; Weng et al., 2016; Xie et al., 2023), liver cancer (Hep3B and HepG2 cells) (Kim et al., 2021; Peng et al., 2024; Wu Z.-c. et al., 2022), leukemia (K562, K562/A02 and KBM5) (Cai et al., 2014; Pal et al., 2010; Yin et al., 2023), pancreatic cancer (PANC-1, AsPC-1, BxPC-3 and CFPAC-1 cells) (Khan et al., 2012; Zhang et al., 2018; Zhang et al., 2021), cholangiocarcinoma (SNU478 cells) (Kim et al., 2024), prostate cancer (LNCaP, PC-3 and DU145 cells) (Chen et al., 2018; Rasul et al., 2013a), gastric adenocarcinoma (SGC-7901 cells) (Rasul et al., 2013b), head and neck squamous cell carcinoma (UM-SCC-10A) (Wang et al., 2015; Wu et al., 2013), osteosarcoma (U2OS, MG-63 and Saos-2 cells) (Di et al., 2014), cervical cancer (HeLa cells) (Liu et al., 2020; Venables et al., 2016), lung squamous carcinoma (SK-MES-1) (Jin et al., 2017), human esophageal cancer (ECA109, EC9706, TE-1, TE13 and KYSE30 cells) (Lu et al., 2018; Wen et al., 2018), glioblastoma (U87, U251, U118, and SHSY-5Y cells) (Xing et al., 2019), endometrial adenocarcinoma (HEC-1 and HEC-1-B cells) (Hu and Yang, 2022; Liu et al., 2020), colorectal cancer (HCT116 and SW620 cells) (Li et al., 2022), gallbladder cancer (NOZ, EH-GB1, SGC-996 and GBC-SD cells) (Lv et al., 2023), and melanoma (B16F10 and A375 cells) (Li et al., 2024). Even in cisplatin-resistant ovarian A2780^cisR^ and SNU-8^cisR^ cells and imatinib-resistant CML KBM5^T315I^ and K562R cells, ISA could also notably inhibit the growth and induce apoptosis (Chun, 2023; Yin et al., 2023). Furthermore, some of these *in vitro* effects (such as in pancreatic cancer, glioblastoma, melanoma, gallbladder cancer, colorectal cancer, prostate cancer and esophageal cancer) have also been confirmed in murine models where tumor volume and weight was reduced while the lipid peroxidation in tumors was increased (Chen et al., 2018; Huang et al., 2021; Khan et al., 2012; Li et al., 2022; Li et al., 2024; Li et al., 2012; Lu et al., 2018; Lv et al., 2023; Wen et al., 2018; Xing et al., 2019; Zhang et al., 2021). ISA can significantly inhibit the cancer growth and induce apoptosis at μM level and the dosages are summarized in Table 5.

**TABLE 5:** ISA inhibits growth and induces apoptosis in various kinds of tumors.

Tumor	Cells/models	Dosage (μM)	Activity/mechanism	References
Breast cancer	MDA-MB-231 cells	5, 10, 15	Apoptosis induction, ROS overproduction, Sirt1 downregulation	Li et al. (2016)
Breast cancer	BT-549	5, 10, 25, 50	STAT3 inhibition	An et al. (2024)
Breast cancer	MCF-7 cells	5, 10, 15	Cell cycle arrest, apoptosis induction, ROS overproduction	Li et al. (2016)
Chronic myeloid leukemia	K562/A02 cells	10, 15, 20	Cell cycle arrest, apoptosis induction, Bcr-Abl downregulation	Cai et al. (2014)
Chronic myeloid leukemia	KBM5 cells	10, 20	Increasing BCR-ABL degradation, lowering survivin expression, apoptosis induction	Yin et al. (2023)
Cholangiocarcinoma	SNU478 cells	5, 10	Apoptosis induction, Hippo-YAP signaling dysregulation	Kim et al. (2024)
Colorectal cancer	HCT116 cells, SW620 cells	5, 10, 20	Cell cycle arrest, apoptosis, autophagy induction	Li et al. (2022)
Endometrial cancer	HEC-1-B cells	5, 10, 20	ROS overproduction, apoptosis induction, MEK/ERK signaling inhibition	Hu and Yang (2022)
Esophageal cancer	ECA109 cells, KYSE30 cells	20, 40, 80	Cell cycle arrest, apoptosis induction, miR-21 downregulation, PDCD4 upregulation	Wen et al. (2018)
Esophageal cancer	ECA109 cells	20, 40	ROS overproduction, extrinsic apoptosis induction	Lu et al. (2018)
Gallbladder cancer	NOZ cells and GBC-SD cells	10, 20, 40	Cell cycle arrest, apoptosis induction, ERK signaling inhibition	Lv et al. (2023)
Gastric cancer	SGC-7901	20, 40	Cell cycle arrest, apoptosis induction, PI3K/Akt pathway inhibition	Rasul et al. (2013b)
Glioblastoma	U87 cells	10, 20, 30	Apoptosis induction, NF-κB/COX2 signaling inhibition, cell cycle arrest	Xing et al. (2019)
Head and neck squamous cell carcinoma	UM-SCC-10A cells	125, 50	Cell cycle arrest, apoptosis induction	Wu et al. (2013)
Liver cancer	Huh7 cells	10, 20, 40	ROS overproduction, apoptosis induction	Peng et al. (2024)
Liver cancer	Hep3B	1, 3, 5	Apoptosis induction, ROS overproduction, JNK signaling activation	Kim et al. (2021)
Liver cancer	HepG2 cells	25, 75, 150	Apoptosis induction, ROS overproduction, Ras/Raf/MEK signaling downregulation	Wu et al. (2022b)
Lung cancer	SK-MES-1 cells	20, 40	Cell cycle arrest, apoptosis induction	Jin et al. (2017)
Melanoma	A375 cells	5, 10, 20	PI3K/AKT/mTOR pathway inhibition, apoptosis induction, cell cycle arrest, autophagy induction, STAT3 inhibition	Li et al. (2024)
Osteosarcoma	U2OS cells	20, 40	Cell cycle arrest, apoptosis induction, ROS overproduction, increasing DR5-FADD interaction, NF-κB p65 inhibition	Di et al. (2014)
Ovarian cancer	OVCAR-3	10, 20, 30, 40, 50	ROS overproduction, cell cycle arrest, apoptosis induction	Xie et al. (2023)
Ovarian cancer	SKOV3 cells	35, 75	Autophagic death induction, cell cycle arrest, PEA15 upregulation	Weng et al. (2016)
Pancreatic cancer	PANC-1 cells	10, 20, 40	Apoptosis induction, inhibition of EGF-PI3K-Skp2-Akt and Wnt pathways	Zhang et al. (2021)
Prostate cancer	PC-3 cells, DU145 cells	10, 20, 40	ROS overproduction, ER stress, apoptosis induction, STAT3 inhibition	Chen et al. (2018)

In most cases, the anticancer activity of ISA is associated with intracellular ROS overproduction. The Micheal-type addition, in which the α, β-unsaturated carbonyl group (as an electrophile) alkylate the biological nucleophiles (such as the SH group in cysteine), was thought to be responsible for the significant antiproliferative action of compounds containing α, β-unsaturated carbonyl, such as alantolactone and ISA (Lawrence et al., 2001; Ma et al., 2013). In eukaryotic cells, ISA can react with the sulfhydryl group of GSH (Figure 1) and decrease the intracellular GSH, incurring an oxidative stress that is toxic to cells (Xie et al., 2023). As GSH is an important antioxidant power in cells, its decrease causes the inability of cells to scavenge excessive ROS that are harmful to cells. This may be the reason why ISA causes ROS overproduction in cancers in so many publications.

The main mechanism underlying its anticancer effects is apoptosis induction, as many other anticancer agents. Intrinsic apoptosis pathway is activated by ISA in many reports (Di et al., 2014; Jin et al., 2017), but some publications also show that ISA can also stimulate extrinsic apoptosis that mediated by death receptor (DR) (Di et al., 2014; Lu et al., 2018; Pal et al., 2010). In ECA109 esophageal cancer cells, ISA significantly increases the expression of death receptor 5 (DR5) and reduces the cleavage of procaspase-10 (due to activation of caspase-10) (Lu et al., 2018). In ISA-exposed Hep3B cells, DR5, DR4 and Fas are also upregulated (Kim et al., 2021). In U2OS cells, DR5, Fas-associating protein with death domain (FADD) and their interactions, were increased by ISA, leading to the activation of caspase-8 (Di et al., 2014). Knockdown of DR5 with siRNA could reverse the ISA-induced viability loss in ECA109 cells, suggesting the involvement of extrinsic apoptosis in ISA-caused cell death (Lu et al., 2018). In this study, intracellular ROS overproduction is also detected, whereas ROS are closely associated with mitochondrial dysfunction and intrinsic apoptosis (Di et al., 2014; Jin et al., 2017; Lu et al., 2018). Although the expression levels of intrinsic apoptosis-associated proteins were not altered significantly in ECA109 cells, ISA did induce intrinsic apoptosis in other cancer cells (Di et al., 2014; Lu et al., 2018; Lv et al., 2023; Wu et al., 2013). In this kind of apoptosis, Bax activation and translocation leads to status alterations of mitochondrial permeability transition pores, facilitating depolarization of mitochondrial membrane potential and subsequent release of cytochrome c and other apoptosis-promoting proteins to cytosol (Kim et al., 2021; Pal et al., 2010). ISA-caused increase in pro-apoptotic Bax is often accompanied by decrease in anti-apoptotic proteins, such as Bcl-2, Mcl-1 and Bcl-xl (Kim et al., 2021; Li et al., 2022; Lv et al., 2023). This contributes to cleavage and activation of caspases, which cleaves PARP (poly (ADP-ribose) polymerase) and mediate apoptosis, as well as inflammation-related pyroptosis (Pal et al., 2010).

ROS in prostate cancer could also induce endoplasmic reticulum (ER) stress mediated through upregulation of p-eIF2α (eukaryotic translation initiation factor 2A) and ATF4. ER stress also promotes apoptosis via increasing the expression of CHOP (CCAAT/enhancer-binding protein homologous protein) (Chen et al., 2018).

In ovarian cancer cells, ISA can reduce the phosphorylation of Akt, which may also contribute to the ROS overproduction and GSH depletion, as well as the downregulation of Bcl-2 (Xie et al., 2023). Inactivation of Akt also retards the cell cycle progression, resulting in arrest at G2/M phase (Li et al., 2024; Xie et al., 2023). Cell cycle arrest is one of the mechanisms underlying the proliferation-inhibitory effects of ISA. G2/M phase arrest (Cai et al., 2014; Di et al., 2014; Khan et al., 2012; Li et al., 2024; Li et al., 2016; Rasul et al., 2013b; Wang et al., 2015; Weng et al., 2016; Xie et al., 2023), S phase arrest (Cai et al., 2014; Di et al., 2014; Khan et al., 2012; Rasul et al., 2013b), and G0/G1 phase (Jin et al., 2017; Li et al., 2022; Lv et al., 2023; Wen et al., 2018; Wu et al., 2013; Xing et al., 2019), have been reported in ISA-treated cancer cells. In the G0/G1 phase arrest, the expression of cyclin D1, CDK2, CDK4 and CDK6 (Li et al., 2022; Lv et al., 2023; Wu et al., 2013; Xing et al., 2019) are decreased by ISA. The lowered expression of cyclin D1 and CDK6 has also been found in G2/M phase arrest caused by ISA, which is associated with decreased expression of CDC2, cyclin B1 and CDK1 (Di et al., 2014; Li et al., 2024; Wang et al., 2015). ISA-induced S phase arrest is found to be associated with reduced levels of cyclin A, cyclin B1 and CDK2 (Cai et al., 2014; Rasul et al., 2013b). Consistent with alterations in cell cycle, the expression of p21, one inhibitor of CDK regulated positively by p53, is increased by ISA (Cai et al., 2014; Li et al., 2022; Wu et al., 2013). Correspondingly, p53, which could be inactivated by Sirt 1 (Silent information regulator 1) that is overexpressed in breast cancer, could also be increased by ISA in several cancers (Li et al., 2016; Wu et al., 2013).

Studies performed by Kim et al (2021) suggested that in Hep3B cells ISA-induced ROS also activate the JNK, one branch of MAPK signaling pathway, resulting in apoptosis mediated by both extrinsic and intrinsic apoptotic pathways where the crosstalk between them is mediated by t-Bid (truncated form of BH3 interacting-domain death agonist).

In addition, ISA also cause cytoprotective autophagy in melanoma and colorectal cancer, through repressing PI3K/Akt/mTOR signaling, besides its apoptosis-promoting effects, where excessive autophagy would lead to death of cancer cells (Li et al., 2022; Li et al., 2024). Autophagic cell death, characterized with upregulation of Beclin 1 and elevated LC3 cleavage, is also induced by ISA in ovarian cancer cells, which was found to be associated with PEA-15 upregulation and following ERK activation (Weng et al., 2016). These indicate the intriguing role of ISA-induced autophagy in its cell death-stimulating effects, which needs further deep exploration.

Besides autophagy, the PI3K and its downstream Akt also regulate cell growth and proliferation. In gastric and ovarian cancers, ISA can inhibit PI3K/Akt signaling, leading to lowered ratio of Bcl-2 to Bax that facilitates apoptosis (Chun, 2023; Rasul et al., 2013b).

In cancers that are closely associated with inflammation, such as glioblastoma, breast cancer and osteosarcoma, NF-κB activation was also inhibited by ISA, and so were the upstream IκB kinase (IKK) and IκB, and downstream COX2 (Di et al., 2014; Wang et al., 2016; Xing et al., 2019). The oncogenic transcriptional factor STAT3 that is upregulated in prostate cancer and melanoma, is also closely associated with inflammation. The phosphorylation of Tyr 705 of STAT3 was also found to be inhibited by ISA in prostate cancer, breast cancer and melanoma (An et al., 2024; Chen et al., 2018; Li et al., 2024). In esophageal cancer, ISA can inhibit miR-21, which silences programmed cell death 4 (PDCD4) and promotes cell proliferation, resulting the unleashing of PDCD4 and cell death (Wen et al., 2018).

The migration and invasion of multiple cancer cells could also be inhibited by ISA treatment (Lv et al., 2023; Wang et al., 2016; Wu Z.-c. et al., 2022; Zhang et al., 2021). The increased expression of matrix metalloproteinase 2 (MMP2) and MMP9, regulated by MAPK and often found in metastatic cancers, could so be suppressed by ISA (Wang et al., 2016). In this context, ISA can also inhibit p38 MAPK and downstream NF-κB phosphorylation (Wang et al., 2016). Another biological process associated with invasion and metastasis is epithelial-mesenchymal transition (EMT), in which vimentin and N-cadherin play positive roles. ISA was shown to inhibit the expression of vimentin and N-cadherin but increase the level of E-cadherin (loss of which is closely associated with EMT) in gallbladder cancer (Lv et al., 2023). Accordingly, the MEK/ERK signaling regulating EMT was blocked by ISA (Lv et al., 2023).

Angiogenesis plays an important role in tumorigenesis, tumor invasion and metastasis, and inhibiting angiogenesis represents a therapeutic direction for cancers. ISA can inhibit the proliferation of human umbilical vein endothelial cells (IC_50_ = 2.5 μg/mL) and the formation of endothelial cell tube *in vitro* (IC_50_ = 1.0 μg/mL), indicating its antiangiogenic potential (Ma et al., 2013). This antiangiogenic activity of ISA has also been confirmed in another report where ISA, at 1 μM, was shown to significantly inhibit the formation of endothelial tube (Zhu Y. et al., 2023). They also showed ISA can inhibit the elongation of intersegmental vessels in zebrafish embryo, although the difference was insignificant at 5 μM and higher concentrations would cause toxicity in these animals (Zhu Y. et al., 2023). To be better used as antiangiogenic agent, ISA has to be modified to lower the toxicity in animals shown by Zhu *et al* (Zhu Y. et al., 2023).

Besides inducing DNA damage, ROS can also activate JNK signaling, as supplementation with ROS scavenger NAC would reduce the phosphorylation of JNK (Wu F. et al., 2022). Through inducing ROS production and activating JNK pathway, ISA can increase the antitumor effects of doxorubicin in colon cancers (thus lowering the cardiotoxicity of doxorubicin) (Wu F. et al., 2022) and the sensitivity of cisplatin in prostate cancer (Huang et al., 2021). ROS accumulation caused by ISA-cisplatin combination can also activate ER stress, as the levels of key related proteins, such as p-eIF2α, ATF4 and CHOP, are increased by this combination (Huang et al., 2021). While in breast tumor cells, ISA also reverses the resistance to doxorubicin of MCF-7/DR cells by 3 fold at 1 μg/mL (Wu et al., 2016). This kind of reversal was due to the decreased efflux and elevated intracellular accumulation of doxorubicin resulting from the inhibition of ABCB1 expression and the reduction in lipid raft stability (Wu et al., 2016). In ovarian cancers, ISA potentiates the cisplatin through a different manner. The increased glycolysis that gives advantage to and is dependent more by cisplatin-resistant ovarian cancers, could be inhibited by ISA, thus increasing the sensitivity of these cisplatin-resistant cells to cisplatin (Chun, 2023). Meanwhile, this combination increases the phosphorylation of JNK and AKT, which regulate cell survival, resulting in enhanced sensitivity of these cells to cisplatin (Chun, 2023). In addition, this combination has shown efficacy in a mouse model of xenograft by two different groups (Chun, 2023; Huang et al., 2021). Besides the capacity to increase the sensitivity of prostate cancer and ovarian cancer to cisplatin (Chun, 2023; Huang et al., 2021), ISA can also increase the radio-sensitivity of head and neck squamous cell carcinoma to radiation through inhibiting ERK1/2 phosphorylation (Wang et al., 2015).

ISA can inhibit the expression of c-myb, a proto-oncogene encoding the transcription factor regulating genes whose products are essential for the cell proliferation, survival and differentiation (Schomburg et al., 2013). Notably, The IC_50_ of ISA for c-myb inhibition (19.23 μM) was lower than that of viability (>30 μM). Given the roles of c-myb in leukemia and other cancers, ISA can be potentially used for developing therapies for these cancers (Ciciro and Sala, 2021; Schomburg et al., 2013).

Many other lactones have antitumor activities, including alantolactone which shows almost similar activities against many tumors as ISA (Liu X. et al., 2021; Wang, 2021). Many derivatives of ISA have been synthesize and their antitumor effects were tested in various kinds of tumors (Anikina et al., 2018; Kumar et al., 2016; Mo et al., 2024; Semakov et al., 2018). Among these derivatives, the most potent one is a hybrid of ISA/hydroxamic acid, ((3αR,4αR,6R,8αR, 9αR)-8α-methyl-3,5-dimethylene-2-oxododecahydronaphtho [2,3-b]furan-6-yl (4-((E)-3-(hydroxyamino)-3-oxoprop-1-en-1-yl) benzoyl)-*R*-alaninate) (Derivative 11, Figure 2K), the IC_50_ of which against the cancer cell lines were below 0.3 μM (Mo et al., 2024). The nanoparticles of this compound are more potent against these cancer cells, and the best IC_50_ is reported to be 0.023 μM against HCT-116 cells (Mo et al., 2024).

## 8 Neuroprotection

Before this part begins, it is noteworthy that ISA can induce detoxifying enzymes such as quinine reductase in Hepa1c1c7 and BPRc1 cells (Lim et al., 2007), indicating the anti-oxidative property of ISA. Although the compound alantolactone and the extract containing both ISA and alantolactone, showed induction of quinine reductase (Lim et al., 2007; Seo et al., 2008), the contribution of ISA should not be excluded. Later, ISA was confirmed by the same group, as well as others, to induce NAD (P) H: quinone oxidoreductase-1 (NQO1), glutathione *S*-transferase, glutathione reductase, γ-glutamylcysteine synthetase and heme oxygenase-1 (HO-1) (Neganova et al., 2019; Seo et al., 2017; Seo et al., 2009). In addition, ISA was shown to be pro-antioxidant in TBARS tests (Neganova et al., 2019). Increased NQO-1, HO-1 and Nrf2 have been demonstrated to be neuro-protective in multiple studies (Chen et al., 2022; Qu et al., 2020). In addition, ISA was shown to suppress ROS production in zebrafish larvae/cells exposed to CuSO_4_ (Zhou et al., 2023). In mice treated with LPS, ISA can increase the expression of Nrf-2 and HO-1, as well as antioxidative enzymes SOD, GPX and CAT, and decrease the MDA content in lung tissues (Yuan et al., 2018), confirming its antioxidative effects *in vivo*.

The production and accumulation of amyloid β peptide (Aβ) in the brain plays important roles in the initiation and development of Alzheimer’s disease (AD) (Chen et al., 2017; Liu X. et al., 2021). ISA can suppress the generation of ROS and superoxide anion induced by amyloid β peptide (Aβ_25-35_) in mouse cortical neurons while elevate intracellular GSH, attenuating the cytotoxicity of Aβ_25-35_ to neurons (Seo et al., 2017). Accordingly, in a mouse amnesia model induced by scopolamine, ISA can decrease the damages of the *cornu ammonis* regions of mouse hippocampus (which is critical in memory and cognition) and alleviate the cognitive impairment, as revealed by Y-maze, passive avoidance and water maze tests; but in Nrf2^−/−^ mice, this kind of improvement has not be seen (Seo et al., 2017). The neuroprotective mechanism may involve the inhibition of acetylcholinesterase, in addition to the activation of Nrf2 by ISA (Seo et al., 2017). The overproduction of inflammatory mediators (such as TNF-α, IL-1β and NO) by microglial cells is involved in the neurodegenerative AD and Parkinson’s disease (PD), as they can damage neurons (Smith et al., 2012; Wang et al., 2018a). In this context, inhibiting neuroinflammation would be beneficial for these diseases. In LPS-stimulated BV-2 microglia cells, ISA can attenuate the neuroinflammation through activating GSK-3β-Nrf2 pathway that subsequently suppresses NF-κB phosphorylation and the generation of inflammatory mediators (Wang et al., 2018a). Derivatives of alantolactone and ISA both show protective effects in the AD model (Ma et al., 2024a; Neganova et al., 2024). The serotonin conjugate of ISA (Derivative 12, Figure 2L) has better protective effects than that of alantolactone (Neganova et al., 2024).

In the pathogenesis of PD that affects one to two percent of all people aged over 65, the loss of dopaminergic neurons in the substantia nigra and excessive neuroinflammation are critical (He et al., 2022; Kim et al., 2015). In PD model of mice induced by MPTP, ISA can inhibit neuroinflammation and damages to dopaminergic neurons mediated by microglial overactivation, and elevate the striatal dopamine (and its metabolites) level, resulting in the reduce in dopaminergic neuron degeneration and improvement of motor dysfunction. The underlying mechanism involves upregulation of Akt/Nrf2/HO-1 signaling and downregulation of NK-κB (He et al., 2022).

The nuclear receptor Nur77 (NR4A1) and Nurr1 (NR4A2) are closely linked to dopamine neurotransmission and mature and function of dopamine neurons, respectively (Kim et al., 2015; Yan J. et al., 2020), suggesting potential targets of treating PD. Nur77 activation has been proposed as potential in treating PD (Liu L. et al., 2021) and Nurr1 agonists can improve the behavioral symptoms and histological abnormalities in PD models (Kim et al., 2015). However, ISA was identified as inhibitors of Nur77 and Nurr1, indicating its neuroprotection was not mediated by Nur77 and Nurr1 (Jung et al., 2019; Munoz-Tello et al., 2020). Recently, Nur77 was identified as a critical mediator recognizing cytosolic LPS and activating NLRP3 inflammasome that leads to release of pro-inflammatory cytokine IL-1β (Zhu F. et al., 2023). In this context, Nur77 inhibition by ISA may help attenuate the neuroinflammation in PD, which needs further confirmation.

## 9 Other activities

### 9.1 Anti-algal activity

ISA also has showed anti-algal activity. In a very early publication, ISA was found to suppress the growth of *Chlorella* cells. However, the respiration rate was largely elevated by ISA (Picman, 1986). With both lowest-complete-inhibition concentration and lowest-observed-effect concentration of 100 μg/mL, ISA can also suppress the growth of *Oscillatoria perornata* and *Selenastrum capricornutum*, two kinds of phytoplanktons in freshwater that could cause cyanobacterial blooms that impact heavily freshwater aquaculture (Krapcho et al., 2006).

### 9.2 Anti-trypanosomal activity

ISA has anti-trypanosomal activity against both *T. brucei rhodesiense* and *T. cruzi*, which cause African *Trypanosoma brucei rhodesiense* (African sleeping sickness) and American *T. cruzi* (Chagas’s disease) that heavily influence the life of people in tropical areas, with IC_50_ of 23.62 and 22.63 μM, respectively (Schmidt et al., 2002).

### 9.3 Cardiomyocyte protective

In the angiotensin II-exposed H9C2 cells, ISA can increase the expression of Nrf2 and HO-1, enhancing the antioxidant capacity of cells, which helps to resist the detrimental effects imposed by angiotensin II, such as oxidative stress and apoptosis induction. The inhibition of MAPK (p38, JNK, ERK) pathways also contribute to the beneficial effects of ISA on cardiomyocytes, which include lowering the angiotensin II-stimulated increases in atrial natriuretic peptide, brain natriuretic peptide and hypertrophy (Yang et al., 2023).

### 9.4 Anti-diabetic and anti-obesity activity

Of the many diabetes mellitus complications, diabetic nephropathy is common and can cause serious outcomes (Jia et al., 2013). High-level glucose and inflammation are key to the initiation and progress of this nephropathy. In rat glomerular mesangial cells exposed to high glucose (HG) to model diabetic nephropathy, ISA can suppress the HG-induced cell proliferation and activation of NF-κB via blocking the degradation of IκBα. Consequently, the expression and secretion of MCP-1, TGF-β and fibronectin were decreased by ISA treatment at nontoxic concentrations (0.2–10 μM) (Jia et al., 2013). This suggest the potential use of ISA in treating diabetic nephropathy. In addition in mice model of gestational diabetes, ISA-containing *Saussurea lappa* extract help to increase the serum insulin level and improve significantly the reproductive outcomes (i.e., living fetuses, maternal weight and fetal body weight) (Raafat et al., 2019).

ISA was also identified as a strong stimulator of glucose uptake in L6 rat skeletal muscle cells: at concentrations below 5 μM that does not affect the viability, ISA can obviously enhance the basal glucose uptake (Arha et al., 2018). Additionally, ISA (10∼20 μM) was confirmed to inhibit the transactivation (rather than the expression or subcellular location) of Nur77 (a transcription factor important in metabolism and inflammation), and simultaneously activate AMPKα, both of which result in the inhibition of mitotic clone expansion (MCE), which was accompanied by reduced expression of cyclin D1 and cyclin A and elevated expression of p27. These effects, as well as downregulation of PPARγ and C/EBPα (which facilitate the transcription of various pre-adipogenic genes), inhibit adipogenic differentiation and intracellular lipid amassing in cultured 3T3-L1 preadipocytes (Jung et al., 2019). Consistently, in mouse model of obesity induced by high fat diet (HFD), ISA can lower the body weight gain and body fat mass, and improve plasma lipid profiles, suggesting a promising candidate for developing drugs for obesity and relevant metabolic diseases (Jung et al., 2019). However, whether this can be used in clinical for anti-type 2 diabetes therapies warrants further and more work to do.

### 9.5 Antidiarrheal activity

Recently, Qu *et al* found that ISA could suppress the ATP and E_act_-stimulated short-circuit current in HT-29 cells having TMEM16A, a Ca^2+^-activated Cl^−^ channel (CaCC), with IC_50_ of 9.2 μM and 11.8 μM, respectively (Qu et al., 2023). In addition, this current in mouse induced by carbachol could also be inhibited by ISA without affecting colon. In the T84 cells expressing CFTR, the cAMP-stimulated Cl^−^ (short-circuit) current mediated by CFTR could be also suppressed by ISA (IC_50_ = 14.6 μM). Because of the roles of CaCC and CFTR in the diarrhea, ISA was further confirmed to be able to inhibit intestinal peristalsis and secretory diarrhea induced by rotavirus in neonate mice (Qu et al., 2023).

### 9.6 Herbicidal activity

ISA also showed herbicidal activity against etiolated wheat coleoptiles, with an IC_50_ of 22 μM, which was lower than that of a marketed herbicidal (Cardenas et al., 2021). In an earlier report, ISA was found to inhibit the germination rate of several Canadian weeds but did not influence the germination of crops (Picman, 1986). Although the ISA derivatives synthesized by Cardenas et al., 2021 didn’t show activity against etiolated wheat coleoptiles, the derivatives of alantolactone and dehydrocostuslactone synthesized showed phytotoxic activity, with the most potent one being pertyolides C (IC_50_:12 μM).

### 9.7 Estrogenic activity

In an *in silico* screen for ligand-receptor docking analysis, ISA shows an affinity for estrogen receptor α (ERα), and in MCF-7 cells that express ER and are sensitive to estrogen, ISA was demonstrated to induce the transcription of pS2, a gene that can be stimulated by estrogen (Kalachaveedu et al., 2018).

### 9.8 Anti-osteoporotic activity

At non-cytotoxic concentration of 0.5–2 μM, ISA could obviously suppress osteoclast formation induced by RANKL (Receptor activator of nuclear factor-kappa B ligand) without influencing the osteogenesis (Lu J. et al., 2020). This kind of anti-osteoporotic effects were mediated by the downregulation of JNK/p38 MAPK, NF-κB and PI3K-AKT signaling pathways that can be stimulated by RANKL. Furthermore, the bone loss in mice induced by ovariectomy could be abrogated by ISA treatment, confirming its *in vivo* anti-osteoporotic effects.

### 9.9 Antidepressant-like activity

In a mice model of anhedonia-like phenotype of depression induced by chronic social defeat stress (CSDS), the depression-like behaviors in mice, such as decreased sucrose preference, prolonged immobility time (in forced swimming test) could be reversed by ISA, at a dose of 10 mg/kg (Wang et al., 2023). Further exploration found that ISA could lower the plasma levels of IL-6 and TNF-α, which are also closely associated with depression (Wang et al., 2023; Yang et al., 2015). Besides, the decreases in protein levels of GluA1, brain-derived neurotrophic factor (BDNF) and postsynaptic density protein 95 in the prefrontal cortex caused by CSDS in mice could be reversed by ISA (Wang et al., 2023). The authors also found that ISA could restore the abundance and diversity of gut microbiota affected by CSDS, indicating the involvement of gut-brain axis in the effects of ISA on depressive behaviors (Wang et al., 2023). These abovementioned activities are briefly summarized in Table 6.

**TABLE 6:** Other activities of ISA.

Activities	Models	Dosage or IC_50_	Effects/mechanisms	References
Anti-algal	*O. perornata*	LOEC: 100 μg/mL; LCIC: 100 μg/mL	Algal growth inhibition	Krapcho et al. (2006)
Anti-algal	*S. capricornutum*	LOEC: 100 μg/mL; LCIC: 100 μg/mL	Algal growth inhibition	Krapcho et al. (2006)
Anti-trypanosomal	*T. brucei rhodesiense*	IC_50_: 23.62 μM	Viability inhibition	Schmidt et al. (2002)
Anti-trypanosomal	*T. cruzi*	IC_50_: 22.63 μM	Viability inhibition	Schmidt et al. (2002)
Cardiomyocyte protective	H9C2 cells	2, 10, 20 μM	Lowering the ANP, BNP, cardiomyocyte hypertrophy caused by angiotensin II; increasing Nrf2/HO-1 activation	Yang et al. (2023)
Anti-diabetic	Diabetic nephropathy induced by high glucose in rat mesangial cells	0.2, 1, 10 μM	Decreasing MCP-1, TGF-β and fibronectin, inhibiting NF-κB activation	Jia et al. (2013)
Anti-obesity	3T3-L1 cells	10, 15, 20 μM	Adipogenesis inhibition, mitotic clonal expansion inhibition, NR4A1 inactivation, AMPKα activation	Jung et al. (2019)
Anti-obesity	C57BL/6 N mice fed with high-fat diet	20 mg/kg/day	Diminution in body weight, fat mass	Jung et al. (2019)
Herbicidal	Etiolated wheat coleoptiles	IC_50_: 22 μM	Not mentioned	Cardenas et al. (2021)
Anti-osteoporotic	Bone marrow macrophages, RAW264.7 macrophages Ovariectomized mice	0.5, 1, 2 μM; 20 mg/mL	Inhibition of osteoclast formation and bone loss, inhibition of activation of JNK, p38, NF-κB and AKT pathways	Lu et al. (2020a)
Antidepressant-like	chronic social defeat stress -exposed mice	10 mg/kg/day	Depressive-like behaviors improved; abnormalities in gut microbiota improved, butyric acid level in gut re-established	Wang et al. (2023)

## 10 Derivatives

ISA is almost insoluble in water (Lu N. et al., 2020), limiting its use. This would be improved by structure modification. For example, the amino adducts improve the solubility in water and pharmacokinetics, and retain the antitumor activity (Kumar et al., 2016). Up to today, isoalantolactone-tryptamine conjugates (Klochkov et al., 2012), anthracycline antibiotics (daunorubicin and doxorubicin) conjugates (Anikina et al., 2018; Semakov et al., 2018), thiophenol conjugates (Semakov and Klochkov, 2020), selenophenol conjugates (Semakov and Klochkov, 2020), piperazine conjugates (Pukhov et al., 2019), halopyridine conjugates (bromopyridine conjugates and iodopyridine conjugates) (Patrushev et al., 2016), and serotonin conjugates (Neganova et al., 2024) have been synthesized by many researchers. In addition, [3 + 2] cycloaddition processes between ISA and diazocyclopropane have also been fulfilled (Ouled Aitouna et al., 2023). ISA can also form multiple hybrid molecules with quinoline, isoquinoline, caffeine, theobromine and theophylline via its exocyclic α-methylene group (Patrushev et al., 2017; Stepanova et al., 2022), which was the major site for the ISA modifications by most groups (Cárdenas et al., 2021; Guo et al., 2014; Kumar et al., 2016; Lawrence et al., 2001; Patrushev et al., 2017; Semakov et al., 2018; Semakov and Klochkov, 2020; Stepanova et al., 2022). The synthesis of dimethylamino derivative and dialkylphosphonates of ISA were also via this group (Dzhalmakhanbetova et al., 2002). The other exocyclic methylene of ISA can also undergo acidic isomerization to produce epoxyisoalantolactone (Klochkov et al., 2006).

Derivatives are synthesized to optimize the capacity of ISA (for example, to promote glucose intake) or to enhance the water solubility (Arha et al., 2018; Kumar et al., 2016). With so many derivatives, the structure-activity relationship could be inferred. Conjugation with pharmacophoric amines may preserve the antioxidant activity but lose the disturbing effects on mitochondria membrane potential of ISA (Neganova et al., 2019). One of the conjugates significantly lower the influence of toxic insults (such as glutamate and H_2_O_2_) on neuroblastoma cells, indicating the potentials for treating neurodegenerative disorders. Some analogs obtain TGF-β1/Smad3-inhibiting activity in fibroblasts that is absent in ISA, which could be used for treating idiopathic pulmonary fibrosis (Li et al., 2018). The retention of diverse activities in ISA derivatives suggested that the bioactivities of ISA come from not only the α-methylene-γ-lactone moiety, but also other functional groups (Guo et al., 2014; Kumar et al., 2016; Lawrence et al., 2001). Another explanation could be that these adducts may break into lactone and the substrates (for example, secondary amines) of former Micheal-type addition reaction, due to the reversibility of this type of reaction (Lawrence et al., 2001). Under some conditions, the ISA conjugates may have opposite effects to ISA. For example, while ISA inhibits the cytochrome c oxidase in the presence of their substrates (ascorbate/N, N, N′, N′- tetramethyl-*p*-phenylenediamine dihydrochloride), one ISA-serotonin conjugate enhances the mitochondrial complex IV activity and overall respiration function of mitochondria (Neganova et al., 2024). Chemical modification of molecules containing α-methylene-γ-lactone moiety is still an attractive field of medicinal chemistry.

## 11 Conclusion

**FIGURE 3:**
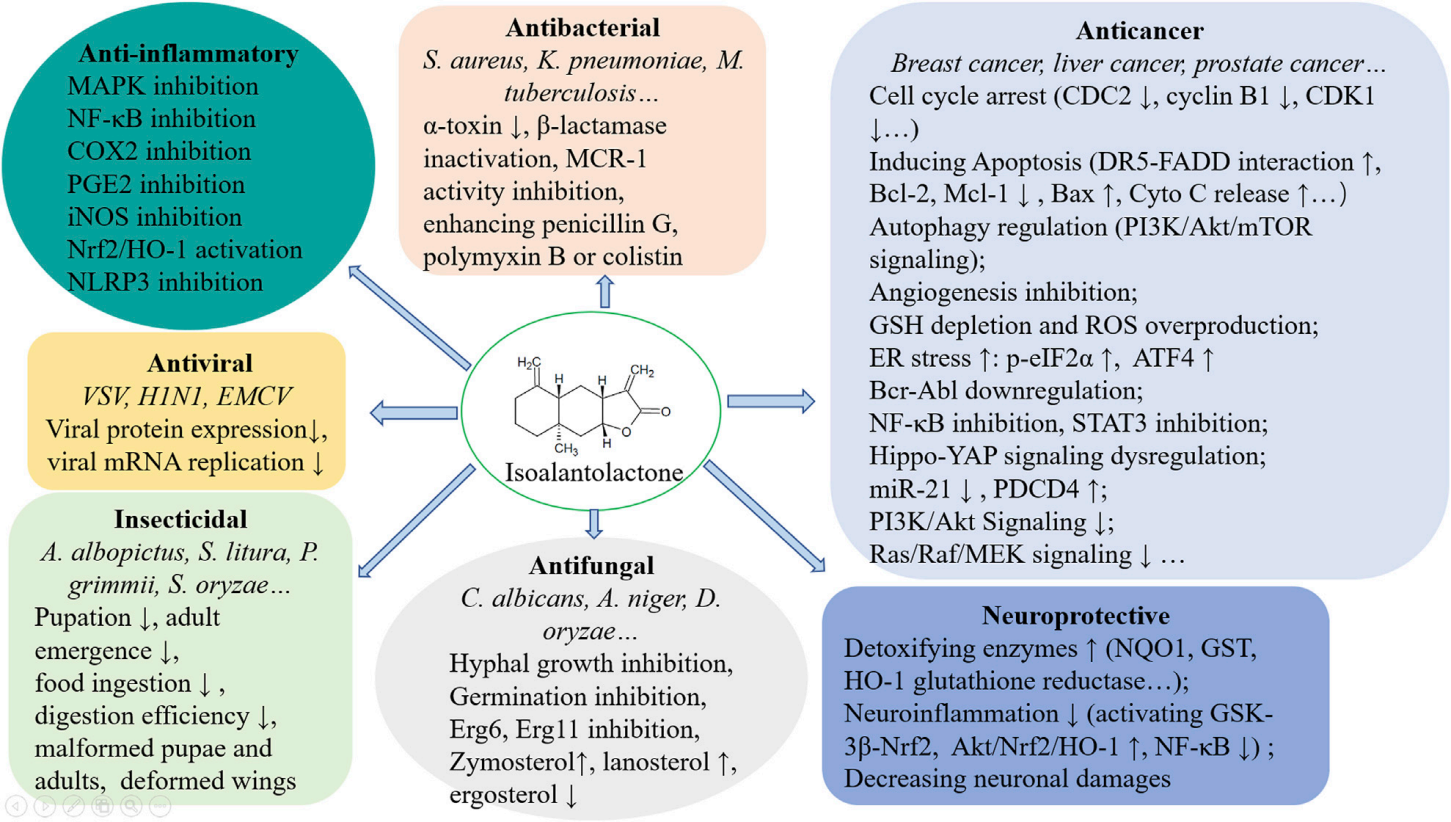
A brief summary of the pharmacological mechanisms of ISA.

ISA is widely distributed in many plants belonging to Asteraceae. In this review, the multiple pharmacological effects of ISA, including antibacterial, antitumor, antifungal, anti-inflammatory, neuroprotective activities, and associated mechanisms were outlined (Figure 3). Lots of further and deep explorations have to be undertaken before these pharmacological activities can be translated into therapeutic options for cancers, microbial infections, inflammatory diseases and neurodegenerative disorders. Total synthesis of ISA has been fulfilled (Tada et al., 1993) and so many derivatives provide a chance to easy analyze the structure-activity relationship, which may further facilitate the structural optimization (to enhance the activity and to lower the toxicity) and development of ISA in drug discovery.
